# 

**Published:** 1912-06

**Authors:** 


### BRISTOL MEDICO-CHIRURGICAL JOURNAL

*PUBLISHED UNDER THE AUSPICES OF*

*THE BRISTOL MEDICO-CHIRURGICAL SOCIETY.*

*Editor:* R. SHINGLETON SMITH, M.D., B.Sc.

WITH WHOM ARE ASSOCIATED

J. MICHELL CLARKE, M.A., M.D.,

J. LACY FIRTH, M.S., M.D., JAMES SWAIN, M.S., M.D.

P. WATSON-WILLIAMS, M.D., *Assistant Editor.*

*Editorial Secretary:* J. A. NIXON, B.A., M.B.

BRISTOL: J. W. ARROWSMITH LTD.

London: J. & A. Churchill, 7 Great Marlborough Street.

*Price One Shilling and Sixpence.*

